# Supplementary material for: Analysis of PPARγ Signaling Activity in Psoriasis
Source: Int J Mol Sci. 2021 Aug 10;22(16):8603. doi: 10.3390/ijms22168603 (PMC8395241; doi:10.3390/ijms22168603)
Supplement: Supplementary file 1 [file ijms-22-08603-s001.zip › Supplemental materials_Analysis of PPARg signaling activity in psoriasis/Pathway models/Models images and html files/Interleukin-17 and interleukin-22 signaling in psoriasis/10232.html]

S100A9


# Protein S100A9

|  |  |
| --- | --- |
| URN | urn:agi-llid:6280 |
| Total Entities | 0 |
| Connectivity | 1908 |
| Name | S100A9 |
| Description | S100 calcium binding protein A9 |
| Notes | The protein encoded by this gene is a member of the S100 family of proteins containing 2 EF-hand calcium-binding motifs. S100 proteins are localized in the cytoplasm and/or nucleus of a wide range of cells, and involved in the regulation of a number of cellular processes such as cell cycle progression and differentiation. S100 genes include at least 13 members which are located as a cluster on chromosome 1q21. This protein may function in the inhibition of casein kinase and altered expression of this protein is associated with the disease cystic fibrosis. This antimicrobial protein exhibits antifungal and antibacterial activity. [provided by RefSeq, Nov 2014] |
| ObjectType | Protein |

---

|  |  |
| --- | --- |
| Pathway | AHR in Intestinal Cell Antimicrobial Barrier Maintenance |
|  | Proteins Involved in Atherosclerosis |
|  | RAGE/AGER and S100 Proteins in Cardiovascular Injury |
|  | IL22 Induces Keratinocyte Proliferation in Psoriasis |
|  | Proteins with Altered Expression in Psoriasis |
|  | Dendritic Cells Function in Psoriasis |
|  | Dendritic Cell Dysfunction in Psoriatic Arthritis |
|  | Immune System Role in Otitis Media |
|  | Proteins Involved in Bronchiectasis |
|  | Proteins Involved in Chronic Obstructive Pulmonary Disease |
|  | Myeloid Derived Suppressor Cells in Cancer Immune Escape |
|  | IFNG/IFNR Expression Targets |
|  | IL6 Expression Targets |
|  | TNF -> STAT Expression Targets |
|  | TLR4 -> AP-1/EGR1/HIF1A Expression Targets |
|  | IL1A Expression Targets |
|  | VEGFA -> STAT Expression Targets |
|  | dsRNA induces innate immunity and INFs in alveolar cells |
|  | AGER feed forward loop in innate immunity |
|  | 2\_Interleukin-17 and interleukin-22 signaling in psoriasis |
|  | 3\_Arterial wall inflammation and calcification in atherosclerosis |
|  | Interleukin-17 and interleukin-22 signaling in psoriasis |

---

|  |  |
| --- | --- |
| MedScan ID | 6280 |

---

|  |  |
| --- | --- |
| LocusLink ID | 6280 |
|  | 20202 |
|  | 94195 |

---

|  |  |
| --- | --- |
| Alias | S100a9 |
|  | OTTHUMP00000015331 |
|  | LIAG |
|  | calprotectin L1H subunit |
|  | MAC387 |
|  | intracellular calcium-binding protein (MRP14) |
|  | migration inhibitory factor-related protein |
|  | CGLB |
|  | calgranulin B |
|  | S100 calcium binding protein A9 |
|  | S109 |
|  | gagb |
|  | myeloid-related protein 14 |
|  | AW546964 |
|  | OTTMUSP00000032973 |
|  | CAGB |
|  | Migration inhibitory factor-related protein 14 |
|  | MRP14 |
|  | S100 calcium binding protein A9 (calgranulin B) |
|  | Leukocyte L1 complex heavy chain |
|  | bee22 |
|  | S109\_HUMAN |
|  | MIF |
|  | NIF |
|  | P14 |
|  | CFAG |
|  | L1AG |
|  | 60B8AG |
|  | MRP-14 |
|  | protein S100-A9 |
|  | S100 calcium-binding protein A9 |
|  | calgranulin-B |
|  | S100 calcium-binding protein A9 (calgranulin B) |

---

|  |  |
| --- | --- |
| GO ID | 0050786 |
|  | 0035662 |
|  | 0016209 |
|  | 0050544 |
|  | 0005509 |
|  | 0008017 |
|  | 0008270 |
|  | 0006919 |
|  | 0061844 |
|  | 0019730 |
|  | 0006915 |
|  | 0006914 |
|  | 0007267 |
|  | 0098869 |
|  | 0032602 |
|  | 0001816 |
|  | 0042742 |
|  | 0050832 |
|  | 0006954 |
|  | 0045087 |
|  | 0002523 |
|  | 0035821 |
|  | 0070488 |
|  | 0030593 |
|  | 0043312 |
|  | 0035606 |
|  | 0051092 |
|  | 0030307 |
|  | 0050729 |
|  | 2001244 |
|  | 0010976 |
|  | 0051493 |
|  | 0032119 |
|  | 0002224 |
|  | 0030054 |
|  | 0062023 |
|  | 0005737 |
|  | 0005856 |
|  | 0005829 |
|  | 0070062 |
|  | 0005576 |
|  | 0005615 |
|  | 0005654 |
|  | 0005634 |
|  | 0005886 |
|  | 0034774 |
|  | 0046872 |
|  | 0031532 |
|  | 0014002 |
|  | 0035425 |
|  | 0006935 |
|  | 0002376 |
|  | 0030595 |
|  | 0018119 |
|  | 0030194 |
|  | 0002793 |
|  | 0050727 |
|  | 0045113 |
|  | 0006417 |
|  | 0016020 |
|  | 0002544 |
|  | 0045471 |
|  | 0032496 |
|  | 0010043 |
|  | 0006917 |
|  | 0007165 |
|  | 0005515 |
|  | 0004871 |
|  | 0005730 |

---

|  |  |
| --- | --- |
| KEGG ID | hsa:6280 |
|  | mmu:20202 |
|  | rno:94195 |

---

|  |  |
| --- | --- |
| Organism | Homo sapiens {Organism urn:agi-taxid:9606} |
|  | Mus musculus {Organism urn:agi-taxid:10090} |
|  | Rattus norvegicus {Organism urn:agi-taxid:10116} |
|  | Mus musculus |
|  | Homo sapiens |
|  | Rattus norvegicus |

---

|  |  |
| --- | --- |
| Mouse chromosome position | 3 39.91 cM |
|  | 3 43.6 cM |

---

|  |  |
| --- | --- |
| OMIM ID | 123886 |

---

|  |  |
| --- | --- |
| Rat chromosome position | 2q34 |

---

|  |  |
| --- | --- |
| Hugo ID | 10499 |
|  | HGNC:10499 |

---

|  |  |
| --- | --- |
| Human chromosome position | 1q21.3 |
|  | 1q21 |

---

|  |  |
| --- | --- |
| Swiss-Prot Accession | P06702 |
|  | P06702.1 |
|  | P31725 |
|  | Q3UP42 |
|  | P31725.3 |
|  | P50116 |
|  | A0A0H2UHJ1 |
|  | P50116.3 |
|  | D3DV36 |
|  | Q6FGA1 |
|  | Q9NYM0 |
|  | Q9UCJ1 |
|  | Q761U7 |

---

|  |  |
| --- | --- |
| PIR ID | B31848 |
|  | S68242 |
|  | JN0686 |

---

|  |  |
| --- | --- |
| GenBank ID | NC\_000001 |
|  | NM\_002965 |
|  | NP\_002956 |
|  | A12029 |
|  | CAA01002 |
|  | A12031 |
|  | CAA01003 |
|  | A12032 |
|  | CAA01004 |
|  | AF237581 |
|  | AAF62536 |
|  | AF237582 |
|  | AAF62537 |
|  | AL591704 |
|  | CH471121 |
|  | EAW53333 |
|  | EAW53334 |
|  | M21064 |
|  | AAA36326 |
|  | AF086362 |
|  | AK311882 |
|  | BAG34823 |
|  | BC047681 |
|  | AAH47681 |
|  | CR542207 |
|  | CAG47003 |
|  | CR542224 |
|  | CAG47020 |
|  | M26311 |
|  | AAA68480 |
|  | X06233 |
|  | CAA29579 |
|  | P06702 |
|  | NC\_000069 |
|  | NM\_009114 |
|  | NP\_033140 |
|  | NM\_001281852 |
|  | NP\_001268781 |
|  | AC122425 |
|  | AJ250496 |
|  | CAC14292 |
|  | CH466547 |
|  | EDL15109 |
|  | EDL15110 |
|  | AK143826 |
|  | BAE25555 |
|  | BC027635 |
|  | AAH27635 |
|  | BF732133 |
|  | BY112406 |
|  | M83219 |
|  | AAB07228 |
|  | P31725 |
|  | NC\_005101 |
|  | XM\_006232780 |
|  | XP\_006232842 |
|  | NM\_053587 |
|  | NP\_446039 |
|  | AC\_000070 |
|  | AAHX01018654 |
|  | AC097705 |
|  | CH473976 |
|  | EDM00535 |
|  | AB118215 |
|  | BAC82423 |
|  | L18948 |
|  | AAA18214 |
|  | P50116 |
|  | AAHY01029034 |
|  | CAI19494 |
|  | ABBA01049433 |
|  | AABR06018860 |
|  | AC\_000133 |
|  | NC\_018912 |
|  | AC\_000025 |
|  | AMYH02001902 |

---

|  |  |
| --- | --- |
| Swiss-Prot ID | S10A9\_HUMAN |
|  | S10A9\_MOUSE |
|  | S10A9\_RAT |

---

|  |  |
| --- | --- |
| Cell Localization | Secreted |
|  | Cytoplasm |
|  | Cytoskeleton |
|  | Cell membrane |
|  | Cytoplasm, cytoskeleton |

---

|  |  |
| --- | --- |
| Ensembl ID | ENSG00000163220 |
|  | ENSP00000357727.3 |
|  | ENST00000368738.4 |
|  | ENSMUSG00000056071 |
|  | ENSMUSP00000070842.5 |
|  | ENSMUST00000069960.11 |
|  | ENSMUSP00000112843.1 |
|  | ENSMUST00000117167.1 |
|  | ENSRNOG00000011483 |
|  | ENSRNOP00000015351.3 |
|  | ENSRNOT00000015351.5 |
|  | ENSMUSP00000112843 |
|  | ENSRNOT00000015351 |
|  | ENST00000368738.3 |
|  | ENSMUSP00000070842 |
|  | ENSP00000357727 |
|  | ENSMUST00000117167 |
|  | ENST00000368738 |
|  | ENSRNOP00000015351 |
|  | ENSMUST00000069960 |

---

|  |  |
| --- | --- |
| MGI ID | MGI:1338947 |
|  | 1338947 |

---

|  |  |
| --- | --- |
| RGD ID | 620267 |

---

|  |  |
| --- | --- |
| Unigene ID | Rn.6703 |
|  | Hs.112405 |
|  | Mm.2128 |

---

|  |  |
| --- | --- |
| Homologene ID | 2227 |

---

|  |  |
| --- | --- |
| IPI ID | IPI00027462 |
|  | IPI00231262 |
|  | IPI00222556 |

---
